# Pharmacists’ Preparedness to Patients Education at the Time of Pandemic—A Cross-Sectional Study with an Example of SARS-CoV-2 Outbreak in Poland

**DOI:** 10.3390/ijerph17186659

**Published:** 2020-09-12

**Authors:** Magdalena Cerbin-Koczorowska, Magdalena Waszyk-Nowaczyk, Piotr Przymuszała

**Affiliations:** 1 Department of Medical Education, Poznan University of Medical Sciences, 7 Rokietnicka St, 60-806 Poznan, Poland; pprzymuszala@ump.edu.pl; 2 Department of Pharmaceutical Technology, Pharmacy Practice Division, Poznan University of Medical Sciences, 6 Grunwaldzka St, 60-780 Poznan, Poland; mwaszyk@ump.edu.pl

**Keywords:** COVID-19, pandemic, community pharmacy, pharmaceutical services, patient education, health promotion, mystery shopper

## Abstract

Although pharmacy employees’ involvement in patient education has great potential, the extent to which they actually provide cognitive services seems inadequate. Given the overburdening of the healthcare system and limited access to medical services due to the severe acute respiratory syndrome coronavirus 2 (SARS-CoV-2) pandemic outbreak, this study aimed to evaluate the preparedness of Polish pharmacy employees for patient education on the new threat. The study was conducted using the mystery shopper method. Two interviewers phoned 90 randomly chosen community pharmacies throughout Poland and presented some inquiries on the SARS-CoV-2. Pharmacists devoted more time to patients than pharmacy technicians (2:22 vs. 1:54), and the information they provided was significantly more comprehensive (*p* = 0.006). The majority of respondents provided an evidence-based recommendation on prevention, symptoms, and management of SARS-CoV-2; however, the scope of advice significantly varied. Community pharmacy staff often expressed their concern about the lack of time to address patients’ questions adequately. No statistically significant differences were found in recommendations provided by chain and non-chain pharmacy staff. Obtained results seem to confirm the possibility of involving pharmacists in public health activities during a pandemic. Nevertheless, providing proper working conditions and adequate just-in-time learning solutions is crucial.

## 1. Introduction

The availability and frequency with which patients visit community pharmacies in comparison with other healthcare facilities places their employees among the first healthcare professionals contacted by patients with inquiries on health-related issues [1,2,3]. This process is additionally reinforced by the limited access to medical services, which prompts patients to search for other sources of information or medical assistance. Consequently, changing societal expectations influenced the pharmacists’ scope of practice. The scope of contemporary pharmacy practice should not be reduced to dispensing drugs but also involve the pharmaceutical care, sharing pharmacists’ expertise on drugs with patients and other members of the medical team, and broadly understood health education of patients, including the disease prevention and promotion of healthy eating and lifestyle [4,5,6,7]. Studies conducted previously already noted pharmacists’ engagement in patient education on, among others, rational antibiotic treatment, healthy lifestyle, sleep problems, or quitting smoking [2,8,9,10,11]. However, other reports show inadequate involvement of pharmacists in patient health education despite their high recognition of its necessity [12,13,14]. Potential reasons for that discrepancy might include limited time and human resources, lack of remuneration for their additional services, or fears of stepping into the physician’s scope of practice [12,14,15].

Meanwhile, although Polish patients still connect pharmacists mostly with selling medicaments [16], there are reports indicating patients’ positive attitudes and expectations for pharmacists’ advice or information on drugs, smoking cessation, and minor or chronic health conditions, including their screening and prevention [17,18]. The majority of Polish pharmacists also express positive attitudes and readiness to expand the scope of their practice [19,20]. However, at the same time, they seem to have an unclear idea of their educatory role [19]. Moreover, the extent to which such services are actually provided remains unsatisfactory in the opinion of the majority of Polish patients [16]. Currently, there are no cognitive services implemented in Polish pharmacies, e.g., vaccinations or advice on smoking cessation services that would be financed by the public insurer. This can affect the perception of pharmacists’ image as producers or dispensers of drugs, and although they provide expert information about them, most of these activities are still more product-focused than patient-centered.

The Severe acute respiratory syndrome coronavirus 2 (SARS-CoV-2) pandemic outbreak caused an unprecedented burden to the healthcare systems worldwide, additionally limiting the access of patients to medical services. Overworked physicians and other healthcare workers might not be able to admit all patients seeking medical attention, especially in minor cases. Due to the novelty of the SARS-CoV-2, some patients might also search for professional information on the new threat. Meanwhile, pharmacists already played a significant role during the 2009 H1N1 pandemic. Their specialized skill set and knowledge allowed them to provide patient education that may increase vaccination rates but also improve public health emergency response efforts [21].

Taking all of the above into account, this study aimed to evaluate the readiness and preparedness of Polish pharmacy employees to provide their patients with essential information on the SARS-CoV-2.

## 2. Materials and Methods

**Figure 1:**
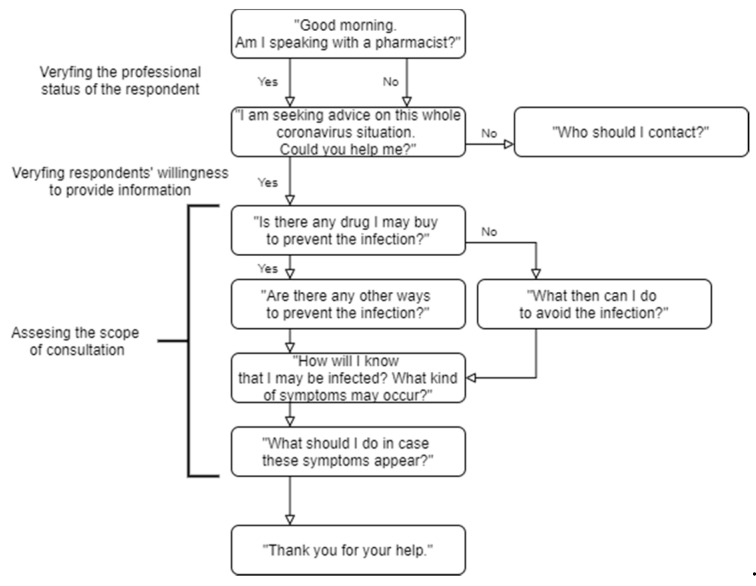
The scheme of consultation scenario which served as a guide for “mystery shopper” consultation.

The study was conducted in March 2020 using the “mystery shopper” (MS) methodology that allows evaluating the performance of healthcare providers [22]. The roles of patients were portrayed by the first and second researchers, both female in their mid-thirties and with previous experience in interviewing pharmacists, telephone interviewing, and the MS method. Data were collected within four working days according to the structured scenario designed by the main researcher and reviewed by two other community pharmacists. Due to the dynamic situation, efforts were made to collect data without unnecessary delays to avoid the potential impact of rapidly changing variables on the collected data. In order to avoid a scenario bias described previously [23], we adapted one scenario that was used by both MS. The consultation scenario and the data collection form were previously discussed by interviewers to minimize the researcher bias. The structured scenario involved MS presenting some concerns about the SARS-CoV-2 outbreak followed by asking the pharmacist about prevention, symptoms, and management of the SARS-CoV-2 infection. The scheme of consultation scenario was presented in Figure 1.

Interviewers phoned a sample of 90 randomly chosen community pharmacies throughout Poland, respecting the proportion between the number of pharmacies in different provinces. The detailed characteristics of pharmacies whose employees provided the consultation are presented in Table 1. 

Answers provided by pharmacists were noted directly during the interview with the use of a data collection form prepared according to the recommendations issued by the Polish Ministry of Health [24] and entered into Microsoft Excel 2016 (Microsoft Company, the United States). Data were further analyzed with the U-Mann–Whitney test and Fisher’s exact test (two-tailed), as appropriate, using the Statistical Software (version 13.3) (TIBCO Software Inc., Palo Alto, USA). For 2 × 4 tables, the Freeman–Halton extension of the Fisher exact probability test was applied using the VassarStats website [25]. Statistical significance was set at *p* < 0.05.

Whether the pharmacist who provided the consultation was employed in a chain or a non-chain pharmacy was verified using the registry of pharmacies [26]. Due to its volume of more than 22,000 entries, an authorial code in Python was used. A chain was defined as five or more pharmacies having the same owner [27], which was confirmed comparing their Taxpayer Identification Numbers (in Polish: Numer Identyfikacji Podatkowej, NIP). The study protocol was approved by the Institutional Review Board.

## 3. Results

In 54 pharmacies, the phone was answered at the first attempt. In 12 pharmacies, no one answered despite three attempts made with at least 10 min intervals. Out of the total 78 phone calls, 59 were conducted with pharmacists (PH) (84.75% female) and another 19 with pharmacy technicians (T) (78.95% female). Seven interviewees were initially not willing to talk, but in the end, they agreed to provide the MS with the information needed. Nine respondents (nPH = 5, nPT = 4) refused to provide the consultation. Among respondents who refused to provide the consultation, three were employed in large towns, three in medium-size towns, one in a small town, and two in villages. All of them were employed in non-chain pharmacies. 

The differences in the willingness of employees to provide the consultation were not statistically significant for both the town size (*p* = 0.6890) and the chain or non-chain pharmacies (*p* = 0.1078). No statistically significant differences were observed between the respondents who provided and refused to provide the consultation in terms of their gender (*p* = 0.1671) and whether they were a pharmacist or a technician (*p* = 0.2097). 

The leading cause of the refusal (66.67%) was the lack of time to give full information resulting from the need to help patients who were present in the pharmacy at the time of the consultation. However, refusing respondents usually indicated the possibility of obtaining reliable information at the Ministry of Health and Chief Sanitary Inspectorate websites. A total of 69 consultations was provided by pharmacy staff (nPH = 54, nPT = 15). The average time of single consultation was 2 min and 16 s (range from 1:04 to 3:50). Ratios and frequencies were employed as descriptive analyses of the results and presented in Table 2 for answers provided by pharmacists and pharmacy technicians. 

Keeping a safe distance was the most frequently recommended way of lowering the risk of contracting coronavirus, followed by hand hygiene. In response to this question, more detailed information was generally provided by pharmacists than pharmacy technicians.

The majority of pharmacists and pharmacy technicians correctly listed the most common symptoms of the COVID-19 infection. Fever was indicated most often. Pharmacists statistically more often mentioned breathing difficulties as one of the symptoms (*p* = 0.033).

In response to the question “What should do in case of those symptoms appear?” both groups most often recommended contacting the Chief Sanitary Inspectorate. The second most common answers were in the case of pharmacists a local isolation ward and in the case of technicians the National Health Fund hotline. Contrary to the Ministry’s recommendations, some respondents also recommended visiting or contacting an emergency ward or calling the emergency number.

No statistically significant differences were found in recommendations provided by chain and non-chain pharmacy staff.

## 4. Discussion

The results of this study demonstrate the readiness and preparedness of most pharmacists and pharmacy technicians to provide patient education during the pandemic outbreak. Most respondents managed to answer MS’s questions satisfactorily, providing at least half of expected correct answers, which signifies the potential of their involvement in similar situations. Some differences, however, were observed between the quality of the information provided by pharmacy employees depending on their qualifications.

In Poland, pharmacy staff may consist of either Master of Pharmacy (pharmacists) or pharmacy technicians. Pharmacists, in order to obtain the license to practice their profession, must complete 5years of studies, followed by a six-month work placement, which is obligatory in order to graduate. Pharmacists are also obliged to improve their qualifications in the course of further professional work as part of continuous training. On the other hand, the diploma of a pharmacy technician is obtained after 2 years of training, after which they begin a 2-year professional internship.

Sancar et al. [23] also compared the quality of advice provided by different employees of community pharmacies. Although there were no significant differences between the number of pharmacists and pharmacy technicians who provided the consultation, the advice of pharmacists was more comprehensive.

Better preparation of pharmacists to be involved in patient education seems to be a reflection of differences in curricula. The profile of the pharmacy graduate assumes using their knowledge and skills in areas related to disease prevention as well as their readiness to promote pro-health behavior [28]. On the other hand, the professional qualifications of pharmacy technicians concern the “preparation and production of medicinal products and trading medicinal products (...) in a pharmacy” [29].

The relationship between the level of education of pharmacy staff and the quality of patient counselling was identified in previously conducted studies [30]. Noteworthy, in the analysis conducted by Koehler and Brown [31] in 67 countries, it was shown that only a small percentage of pharmacy-technician learners are satisfied with the quality of their education. In our study, some shortcomings and deficiencies were observable during the consultations. For instance, some of the provided information was inconsistent with the Ministry’s recommendations. This may be due to the fact that the SARS-CoV-2 is a new virus, and thus, reliable information on it was not as easily accessible as in the case of other diseases. The extension of the average working time of pharmacy employees resulting from reduced access to professional staff [32] makes it even more difficult to find enough time to fill knowledge gaps.

On 11 March 2020, a week after the first case of coronavirus was detected in Poland, the Supreme Pharmaceutical Chamber published on its website [33] educational materials for pharmacists, including guidelines developed by the Chief Sanitary Inspectorate. The currently existing one-way communication paths between the pharmacy chambers and pharmacies make it difficult to verify to what extent the professional staff got acquainted with the available materials. 

It may be worth considering setting up a central LMS (learning management system) platform enabling effective management of the educational process in accordance with the qualifications of pharmacy employees. A systematic review performed by Salter et al. [34] on the topic of e-learning use for continuing pharmacy education confirmed an increase in knowledge immediately after training, regardless of the topic and form. The same observations in the Polish study group were made by the team of Nesterowicz et al. [35], also confirmed by high satisfaction among participants. 

Currently, the majority of educational activities intended as part of continuous training, although aimed at maintaining high-quality patient care, are organized outside the working context. Meanwhile, Coppus et al. [36] proved the use of distance learning tools in pharmacy education allows *“to provide just-in-time learning through on-the-job-training, with the potential for teaching and learning to directly impact on practice”.* Moreover, the use of remote tools allows to quickly reach learners who live in geographically diverse areas [34].

Our results also show that the complexity of the advice provided varies greatly between the members of the study group. In some cases, respondents provided only one answer to the given question. However, it should be emphasized that in Poland, pharmacy employees still rarely provide such cognitive services other than giving drug-related advice. It seems that educational materials for pharmacists should, therefore, be more orientated to form a dedicated consultation pathway, similar to one designed by Amariles et al. [37].

Despite the positive attitudes of patients and doctors concerning the involvement of pharmacists in patient education [18,38], previous studies may suggest pharmacists’ low self-esteem in this area (unpublished results). Despite the aforementioned advantages of e-learning, its usefulness in shaping attitudes and skills is limited. Meanwhile, the use of simulated-patient (SP) methods allows for maintaining the benefits of a work-based context while focusing on skills and competency domains. The SP method involves re-enacting a previously designed scenario with the participation of a pharmacist-learner and an actor in the role of a patient. It was proven that the implementation of this method in the pharmacy curriculum could effectively build self-confidence and improve the comfort of working with patients [39]. Unfortunately, the use of the SP method in teaching future pharmacists in Poland is still limited.

Another striking result to emerge from the data is that many representatives of pharmacy staff expressed their concerns about the lack of time to address patients’ questions adequately. Polish media are currently raising the alarm about the decreasing number of available pharmacy staff, with a simultaneous abrupt increase in the number of patients [40]. Previously conducted studies from various countries, including Poland, show that despite pharmacists’ openness to involvement in the provision of cognitive services, including patient health education, they often mention the lack of time, among others, as barriers to its popularization [41,42]. Moreover, at the time of difficulties in access to primary care physicians, pharmacists are taking on further responsibilities related to, for example, issuing prescriptions for Rx drugs. Eades et al. [6] indicated that pharmacists often view their traditional role of drug dispensing as more important than engaging in health education. The majority of respondents in our study, however, provided at least primary, evidence-based recommendations. However, pharmacy managers should be encouraged to delegate administrative work to pharmacy technicians and to take advantage of pharmacists’ intellectual potential for patient education and counselling.

Available evidence supports the value of professional pharmacy services, including patient education. Ung [43] even stated that *“community pharmacists have a key role in preventing the spread of the 2019-CoV virus. They are charged with key responsibilities of informing, advising and educating the community (...)”*. Nevertheless, the results of studies conducted at the time when the cognitive services were being popularized in Australia and the USA indicated that broadening the range of duties should be accompanied not only by training but also by providing pharmacists with proper working conditions and remuneration [44]. 

Meanwhile, in the initial phases of the pandemic, the Polish authorities expected pharmacy owners to ensure the safety of pharmacists while performing their professional duties. Only after the intervention of the Pharmaceutical Chamber, the government support was extended on community pharmacies staff [45]. Similarly, when the government decided to reimburse flu vaccines for healthcare professionals, community pharmacists were not initially included in this group [46]. These situations seem to suggest that Polish policy-makers do not perceive pharmacists on a par with other healthcare representatives.

On further analysis of the working conditions of community pharmacists, several other factors significantly impeding the reform of pharmacy practice can be identified. Firstly, although pharmaceutical care was legally introduced in 2008 [47], there is a lack of precisely identified scope of services that could be realized in it. Despite successive teams of experts for the implementation of pharmaceutical care in Poland, still, no measurable actions have been taken [48].

Secondly, despite the ongoing process to pass the Pharmacy Profession Act, which would regulate the pharmacist’s rights and duties related to the provision of patient-centered care, so far, nothing has been established [49].

The resistance of representatives of other healthcare professions also seems to be not without significance, which may result from the physician-centered model of healthcare in Poland [50]. Many physicians are convinced that pharmacists control their work while, in fact, they support their actions and increase the safety of patients’ pharmacotherapy [51].

Finally, the issue of remuneration cannot be ignored. The data for 2019 show that the average net salary of a Master of Pharmacy in Poland ranged from PLN 3500 in non-chain pharmacies to PLN 3800 in chains with more than 30 pharmacies [52]. Despite the fact that pharmacists stand on the front lines during the pandemic, any reported increases in their salaries are related only to the attendance bonuses [53]. It is also worth noting that although pharmaceutical services bring many economic benefits and savings for health care, the National Health Fund still does not make decisions in this regard [54,55].

### Study Limitations

The findings of this study have to be seen in the light of some limitations. Due to the dynamically changing epidemiological situation, the researchers decided to collect data as quickly as possible to avoid the influence of other variables on the results obtained. As a result, data were obtained from a limited number of respondents, which prevents their generalization to the entire study population. For the results to be representative on a national scale, the study group should be enlarged to a representative number of 379 community pharmacies [56].

Coronavirus is a new health challenge for the entire world. Therefore, access to reliable knowledge sources in the first months of the pandemic was significantly limited. Consequently, it would be worthwhile to evaluate the sources from which healthcare workers, including pharmacy staff, obtain professional knowledge.

Moreover, due to the SARS-CoV-2 outbreak, it was not possible to conduct this study using the classic MS in community pharmacy visits. In order to ensure the maximum safety of the interviewers, it was decided to conduct the survey using telephone calls. For this reason, in this study, we focused on the content of the consultation. It seems valuable to supplement the data with the assessment of pharmacy staff’s non-technical skills that may affect the effectiveness of patient education.

## 5. Conclusions

Pharmacists are willing to engage in patient education activities during a pandemic. However, it is crucial to reorganize their working conditions so that they can use their intellectual potential to care for the patient without neglecting other duties. Efforts should also be made to introduce appropriate just-in-time learning solutions to provide pharmacy employees with up-to-date, evidence-based knowledge, especially in case of a new threat or crisis.

## Figures and Tables

**Table 1:** Characteristics of randomly chosen community pharmacies.

Characteristic		*n* (%)
Province [in Polish] (total number of community pharmacies)		
	Lower Silesian [dolnośląskie] (991)	6 (8.70)
	Kuyavian-Pomeranian [kujawsko-pomorskie] (611)	3 (4.35)
	Lublin [lubelskie] (785)	5 (7.25)
	Lubusz [lubuskie] (331)	1 (1.45)
	Łódź [łódzkie] (855)	5 (7.25)
	Lesser Poland [małopolskie] (1073)	6 (8.70)
	Masovian [mazowieckie] (1701)	10 (14.49)
	Opole [opolskie] (310)	1 (1.45)
	Subcarpathian [podkarpackie] (665)	3 (4.35)
	Podlaskie [podlaskie] (387)	2 (2.90)
	Pomeranian [pomorskie] (704)	5 (7.25)
	Silesian [śląskie] (1437)	10 (14.49)
	Holy Cross [świętokrzyskie] (393)	2 (2.90)
	Warmian-Masurian [warmińsko-mazurskie] (418)	2 (2.90)
	Greater Poland [wielkopolskie] (1278)	5 (7.25)
	West Pomeranian [zachodniopomorskie] (560)	3 (4.35)
Exact location ^a^		
	large towns	20 (28.99)
	medium towns	17 (24.64)
	small towns	21 (30.43)
	villages	11 (15.94)
Type of pharmacy ^b^		
	chain	18 (26.09)
	non-chain	51 (73.91)

^a^ Small towns—population below 20,000 inhabitants; medium towns—population 20,000–100,000 inhabitants; large towns—population above 100,000 inhabitants. ^b^ chain ≥ 5 pharmacies under the same brand.

**Table 2:** Recommendations for patients provided by pharmacists and pharmacy technicians.

	Consultations Details			Pharmacists	Pharmacy Technicians	Total ^a^	
Number of consultations				54 (100.00)	15 (100.00)	69 (100.00)	
Average time of a single consultation				2:22	1:54	2:16	
Category	Information Provided by Pharmacy Staff						*p*-value
Prevention	Keep safe distance			51 (94.44)	12 (80.00)	63 (91.30)	0.112
	Wash your hands often			43 (79.63)	9 (60.00)	52 (75.36)	0.174
		alcohol-based liquids/gels		27 (50.00)	4 (26.67)	31 (44.93)	0.146
		water with soap		27 (50.00)	3 (20.00)	30 (43.48)	**0.045**
	Avoid touching eyes, nose and mouth			17 (31.48)	1 (6.67)	18 (26.09)	0.093
	Cover mouth/nose while sneezing/coughing			9 (16.67)	0 (0.00)	9 (13.04)	0.189
		the average number of information provided by a single respondent	Q1	2	1	2	**0.006**
			Q2	3	2	3	
			Q3	6	3	6	
Drug usage	No drug helps to prevent the infection			45 (83.33)	8 (53.33)	53 (76.81)	**0.033**
Symptoms	fever			49 (90.74)	13 (86.67)	62 (89.86)	0.641
	breathing difficulties			45 (83.33)	8 (53.33)	53 (76.81)	**0.033**
	dry cough			36 (66.67)	12 (80.00)	48 (69.57)	0.527
		the average number of information provided by a single respondent	Q1	2	2	2	0.222
			Q2	3	2	2	
			Q3	3	3	3	
Source of aid	Chief Sanitary Inspectorate			40 (74.07)	8 (53.33)	48 (69.57)	0.203
	isolation ward at the local hospital			28 (51.85)	4 (26.67)	32 (46.38)	0.142
	National Health Fund hotline			14 (25.93)	6 (40.00)	20 (28.99)	0.341
	emergency ward at the local hospital			4 (7.41)	1 (6.67)	5 (7.25)	0.999
	emergency telephone number (112)			3 (5.56)	0 (0.00)	3 (4.35)	0.999
	local physician’s office			2 (3.70)	1 (6.67)	3 (4.35)	0.527
		the average number of information provided by a single respondent ^b^	Q1	1	1	1	0.141
			Q2	2	1	2	
			Q3	2	2	2	

**^a^** Respondents could choose more than one answer. The results do not add up to 100%. ^b^ including only correct answers. Q—question; A—provided answers; Q1—lower quartile; Q2—middle quartile; Q3—upper quartile. Statistically significant differences at *p* < 0.05 are presented in bold.
